# Supplementary material for: RA3 is a reference-guided approach for epigenetic characterization of single cells
Source: Nat Commun. 2021 Apr 12;12:2177. doi: 10.1038/s41467-021-22495-4 (PMC8041798; doi:10.1038/s41467-021-22495-4)
Supplement: Supplementary file 1 — Supplementary Information [file 41467_2021_22495_MOESM1_ESM.pdf]

Supplementary Information for

A reference-guided approach for epigenetic characterization of single cells

Shengquan Chen[1,2†], Guanao Yan[3,†], Wenyu Zhang[2], Jinzhao Li[2], Rui Jiang[1,*], and Zhixiang Lin[2,*]

[1]MOE Key Laboratory of Bioinformatics, Bioinformatics Division, Beijing National Research Center for Information Science and Technology, Department of Automation, Tsinghua University, Beijing 100084, China
[2]Department of Statistics, The Chinese University of Hong Kong, Sha Tin, Hong Kong SAR, China
[3]School of Mathematical Sciences, Zhejiang University, Hangzhou 310007, China

[†]These authors contributed equally: Shengquan Chen, Guanao Yan
[*]Correspondence should be addressed to: ruijiang@tsinghua.edu.cn, zhixianglin@cuhk.edu.hk

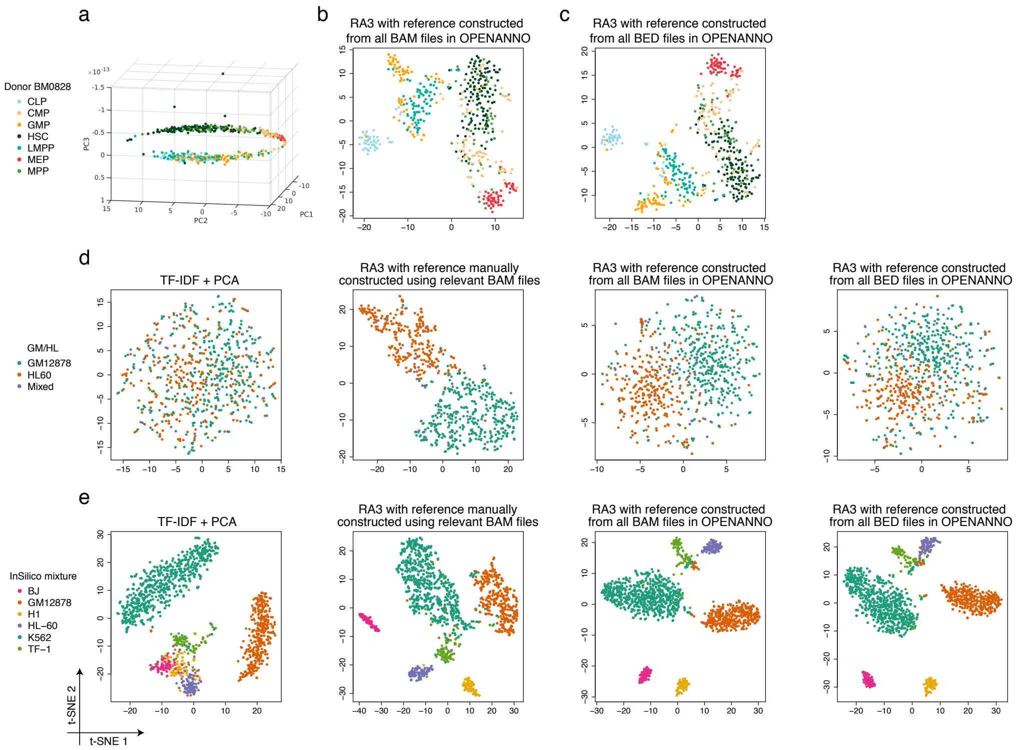

Supplementary Fig. 1 Feature embedding with bulk reference data. **a** Visualization of the cells in the dataset of donor BM0828 using the reference guided approach introduced by Buenrostro et al.[1], that scores each cell by the identified PCs of variation in bulk ATAC-seq samples (note that the input of this approach is the data centered by mean counts in peaks but not TF-IDF transformed), and obtains low-dimensional visual representation by performing PCA on the Pearson correlation matrix of the cell scores. **b** t-SNE visualization of the cells in the dataset of donor BM0828 using latent features obtained from RA3 with reference constructed from BAM files of all the bulk samples in OPENANNO. **c** t-SNE visualization of the cells in the dataset of donor BM0828 using latent features obtained from RA3 with reference constructed from BED files of all the bulk samples in OPENANNO. **d** t-SNE visualizations of the cells in the GM/HL dataset using latent features obtained from TF-IDF + PCA and from RA3 using reference data constructed from different samples, including BAM files of bulk DNase-seq samples of relevant cell lines, BAM files of all the bulk samples in OPENANNO, and BED files of all the bulk samples in OPENANNO. **e** t-SNE visualizations of the cells in the InSilico mixture dataset using latent features obtained from TF-IDF + PCA and from RA3 using reference data constructed from different samples, including BAM files of bulk DNase-seq samples of relevant cell lines, BAM files of all the bulk samples in OPENANNO, and BED files of all the bulk samples in OPENANNO. SAMtools[2], BWA[3], and Python packages including NumPy[4], SciPy[5], pysam[2], and pandas[6] were used to process data. Abbreviations: TF-IDF, term frequency-inverse document frequency transformation; PCA, principal component analysis.

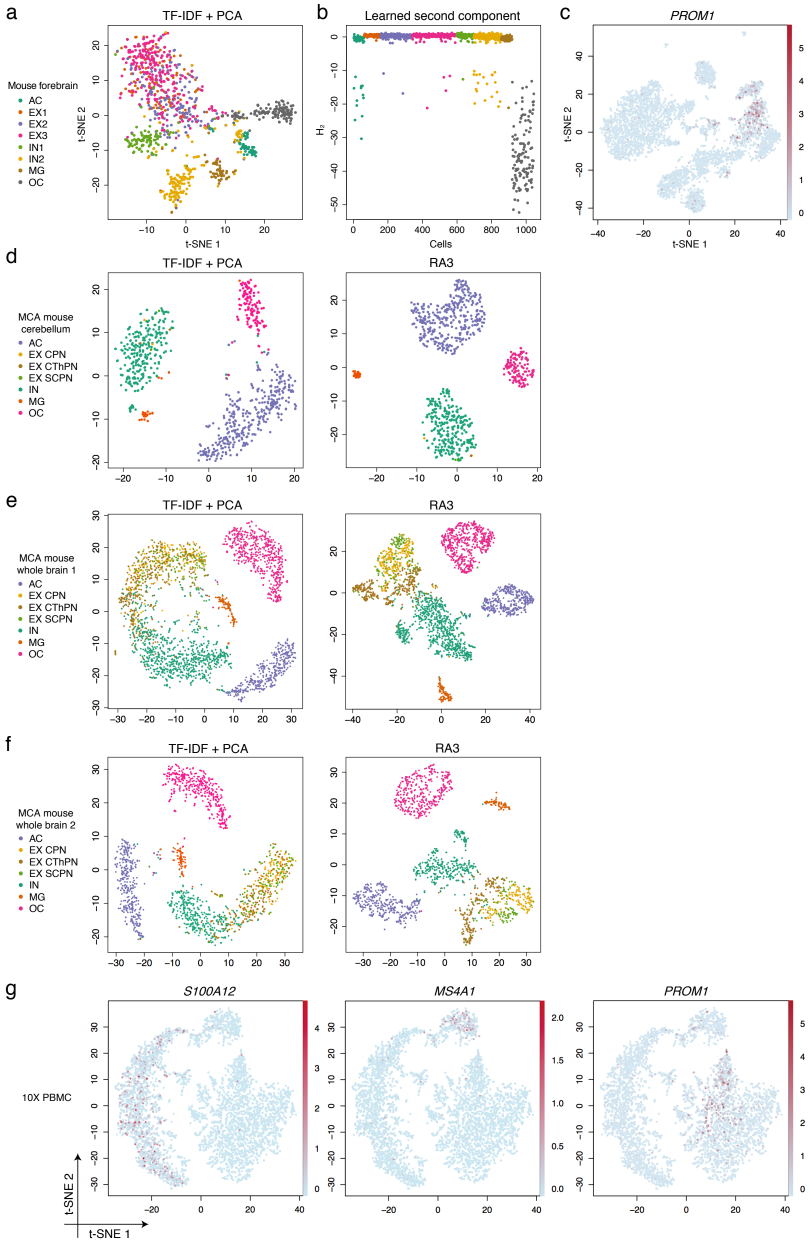

Supplementary Fig. 2 RA3 incorporates pseudo-bulk data as reference. **a** t-SNE visualization of the cells in the mouse forebrain dataset (half) using the latent features obtained from TF-IDF + PCA. **b** Another learned second component with spike-and-slab prior in the mouse forebrain dataset (half), when we implemented RA3 with the imcomplete reference. **c** t-SNE visualizations of cells in the 10X PBMC dataset using the latent features obtained from RA3 with pseudo-bulk reference constructed from another PBMC dataset. Chromatin accessibility of PROM1 (a marker gene of CD34+ cells) is projected onto the visualizations. **d** The dataset of MCA mouse cerebellum. **e** The dataset of the first MCA mouse whole brain sample. **f** The dataset of the second MCA mouse whole brain sample. For the above three MCA mouse brain datasets, we obtained the latent features obtained from TF-IDF + PCA and from RA3 with the pseudo-bulk reference constructed using the mouse forebrain dataset, and then implemented t-SNE for visualization. **g** t-SNE visualizations of the cells in the 10X PBMC dataset using latent features obtained from TF-IDF + PCA. Chromatin accessibility of *S100A12* (a marker gene of monocytes), *MS4A1* (a marker gene of B cells), and *PROM1* (a marker gene of CD34+ cells) are projected onto the visualizations, respectively. Abbreviations: TF-IDF, term frequency-inverse document frequency transformation; PCA, principal component analysis.

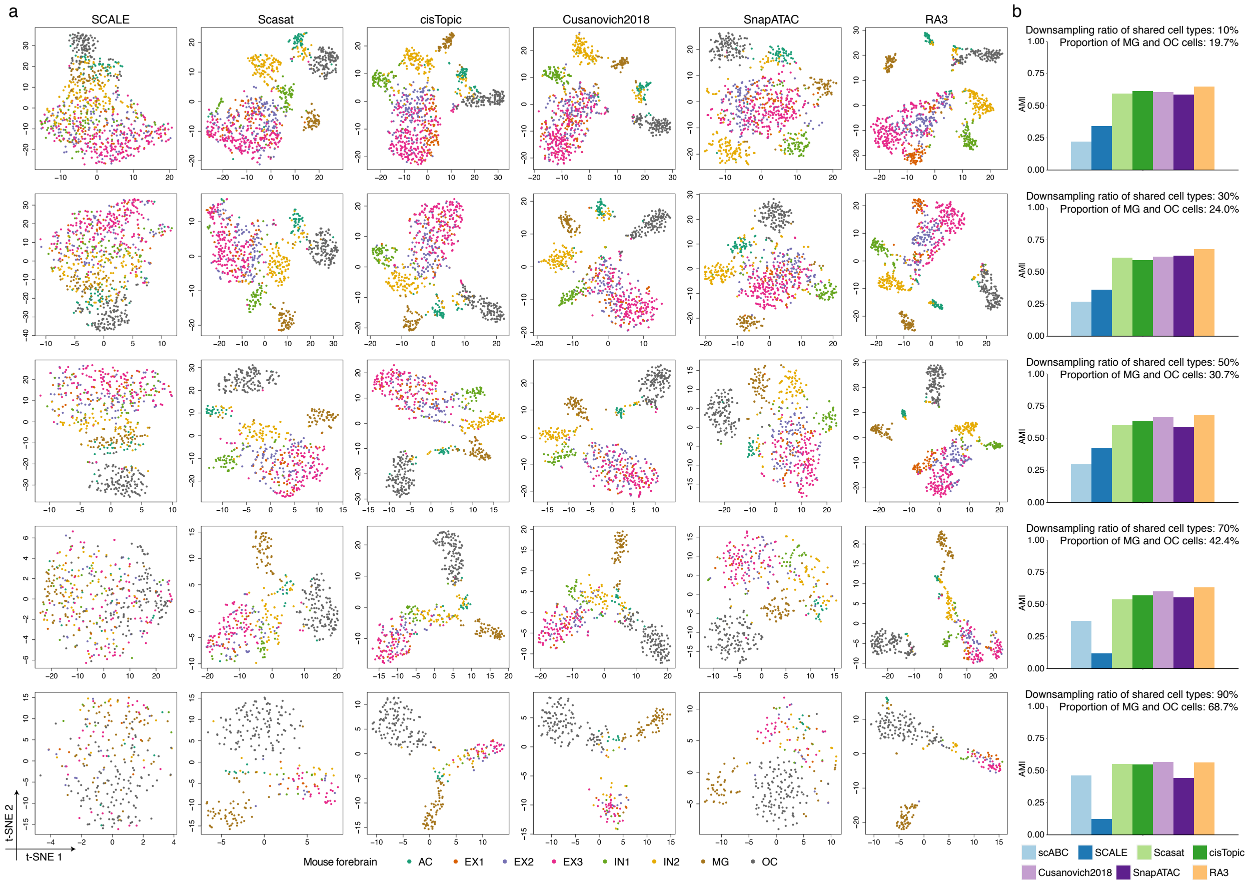

Supplementary Fig. 3 Performance comparison when the overlap between scCAS data and the reference data gradually decreases, using the mouse forebrain dataset (half) and the incomplete reference data (without MG and OC cells). **a** t-SNE visualization of the cells in datasets with downsampling ratios of shared cell types varying from 10% to 90%. **b** The clustering performance using datasets with downsampling ratios of shared cell types varying from 10% to 90%. Abbreviations: AMI, Adjusted Mutual Information.

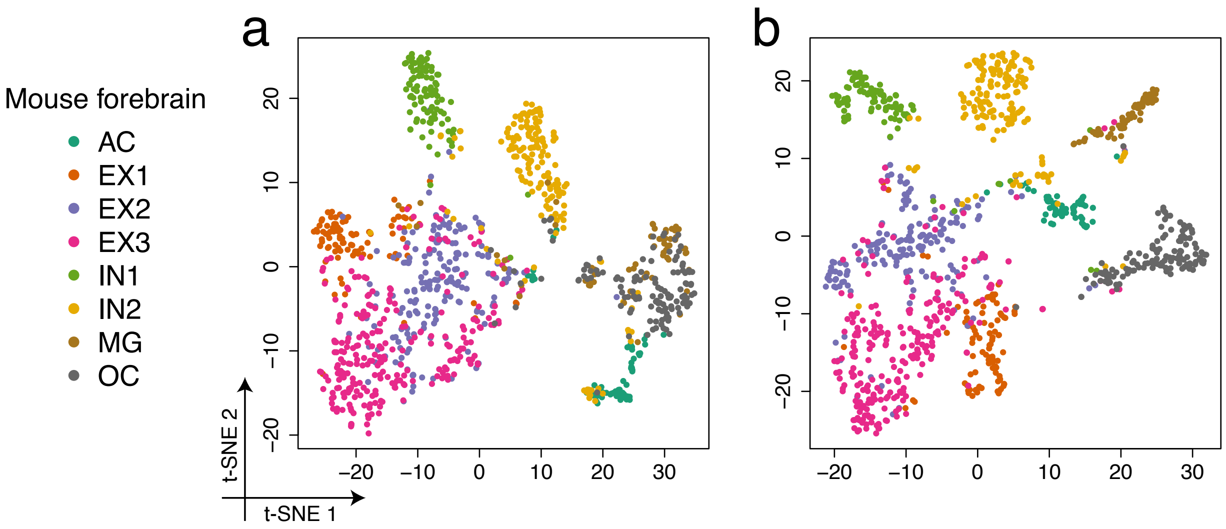

Supplementary Fig. 4 An example demonstrating the iterative peak calling and clustering strategy for better identification of the rare cell subpopulations in target scCAS data. **a** t-SNE visualization of the cells in the mouse forebrain dataset (half) after holding out the MG- and OC-specific peaks. The latent features were obtained from implementing RA3 using incomplete reference data (without MG and OC cells). **b** t-SNE visualization of the cells in the mouse forebrain dataset (half) using peaks obtained by the iterative peak calling strategy. The latent features were obtained from implementing RA3 using incomplete reference data (without MG and OC cells).

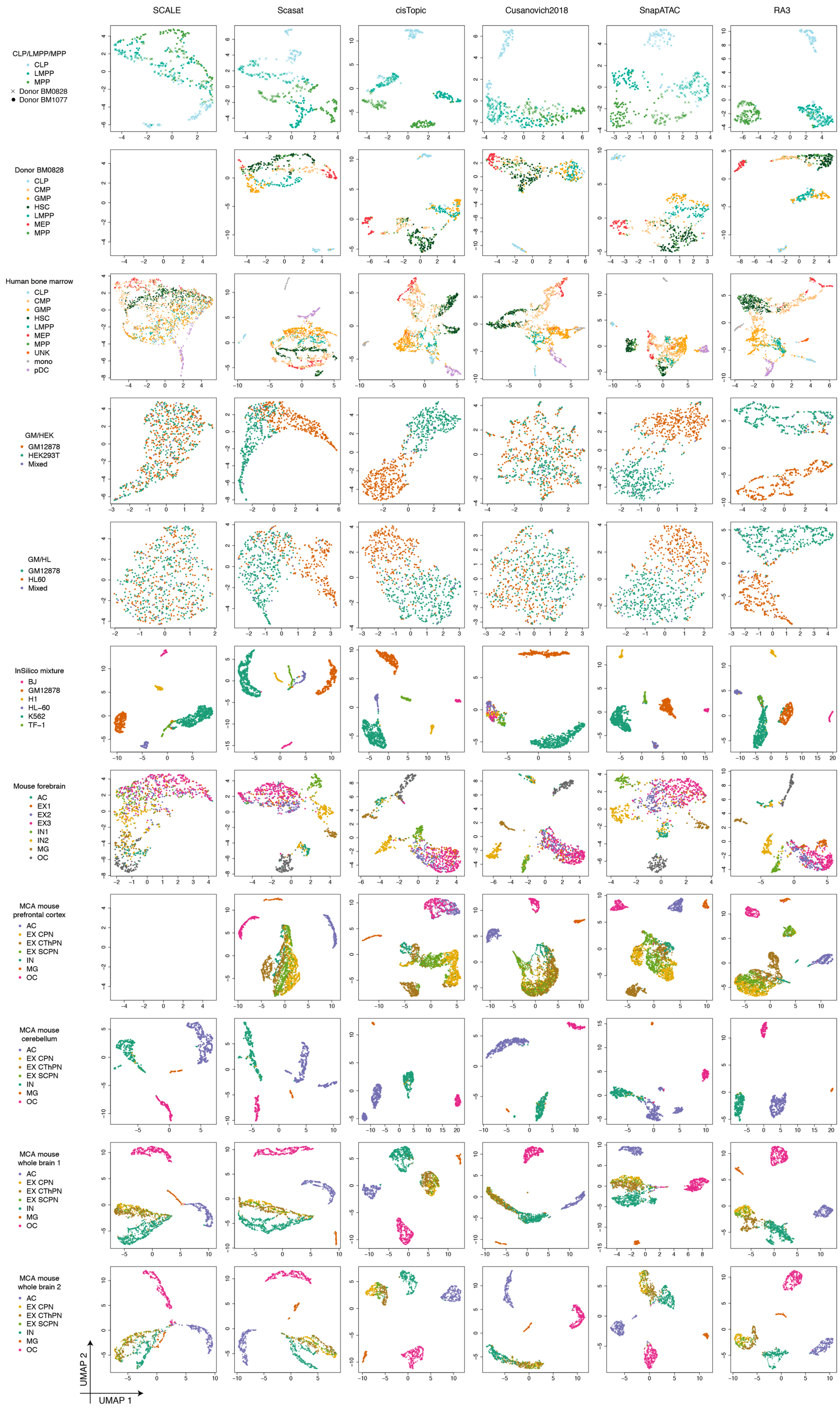

Supplementary Fig. 5 UMAP visualizations of the cells in various datasets using latent features obtained from different methods.

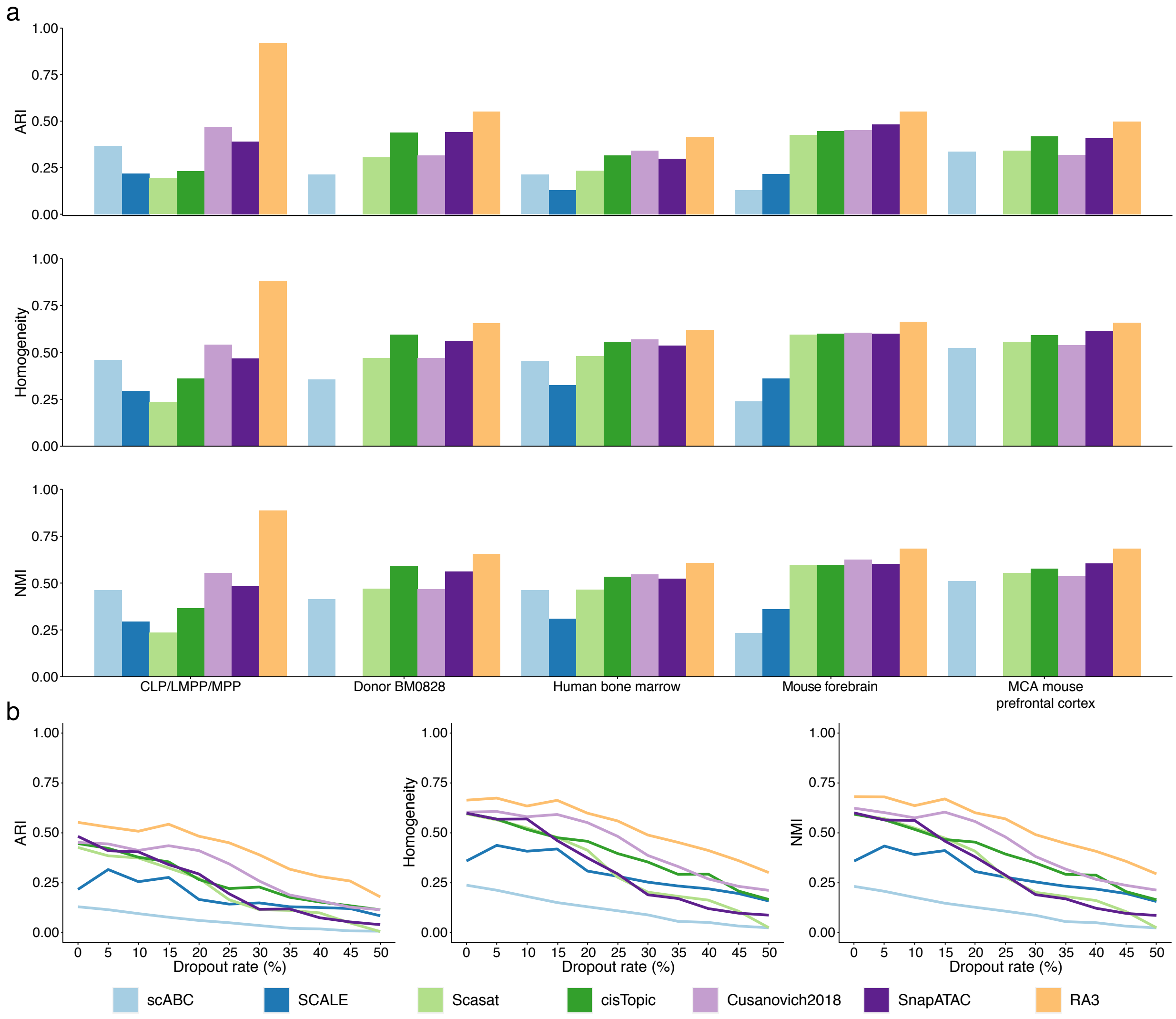

Supplementary Fig. 6 Assessment of the clustering results. We implemented Louvain clustering on the low-dimensional representation provided by each method to get the cluster assignments. The cluster assignments for scABC were obtained directly from the model output. **a** The clustering performance using different methods evaluated by Adjusted Rand Index (ARI), Homogeneity score (Homogeneity), and Normalized Mutual Information (NMI). **b** The clustering performance using different methods on the mouse forebrain dataset (half) at different dropout rates evaluated by ARI, Homogeneity, and NMI. Python package scikit-learn[7] was used to calculate the metrics.

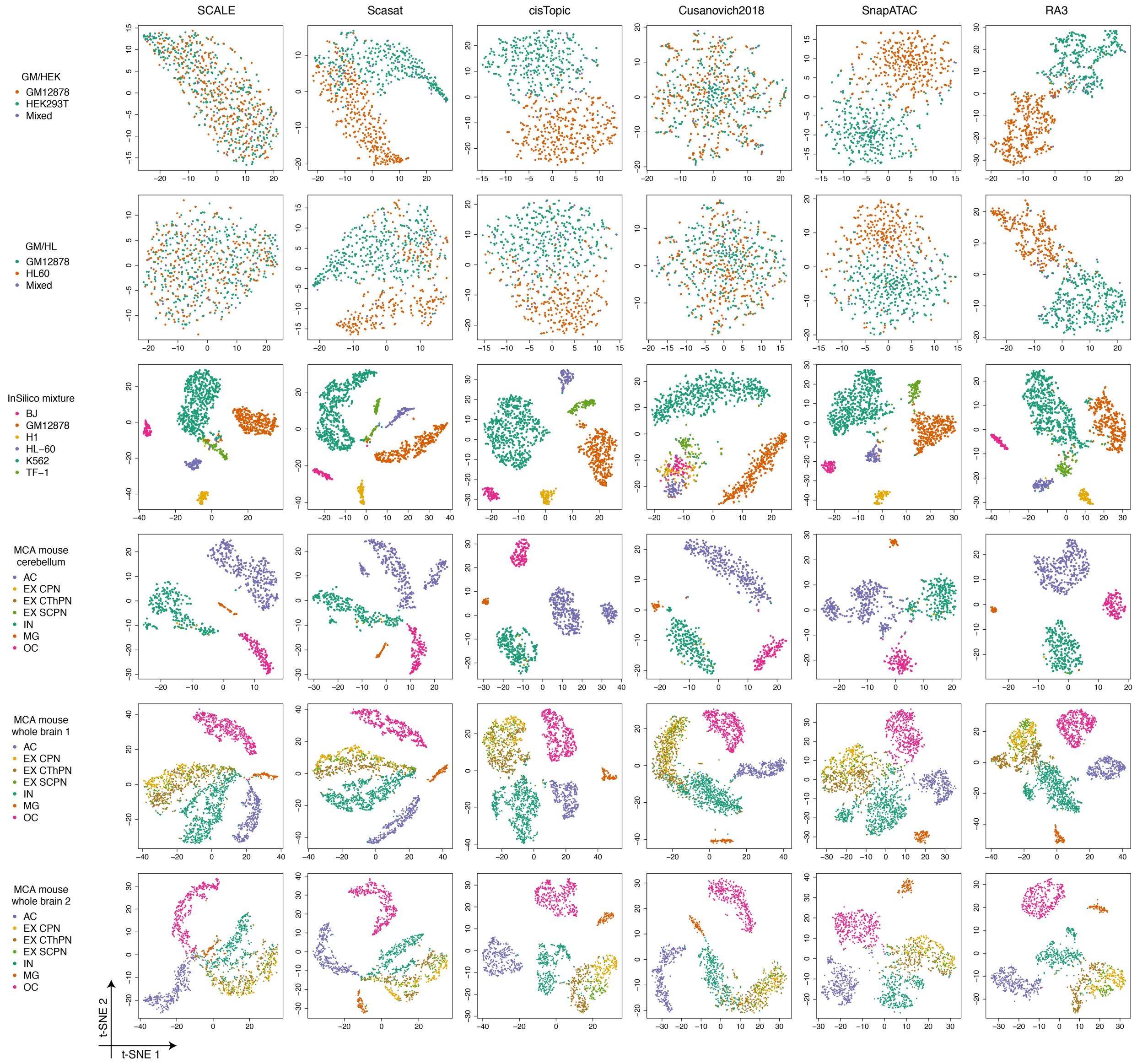

Supplementary Fig. 7 t-SNE visualizations of the cells in the datasets of GM/HEK, GM/HL, InSilico mixture, MCA mouse cerebellum, and two samples of the MCA mouse whole brain using latent features obtained from different methods.

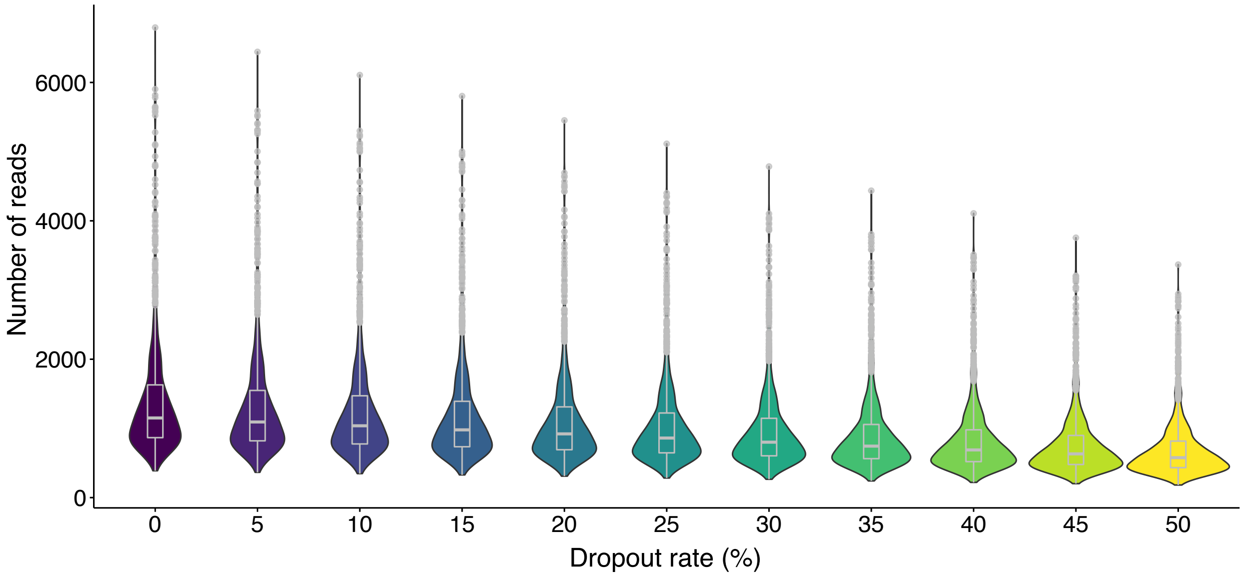

Supplementary Fig. 8 Sequencing depth of the 1043 biologically independent cells in the mouse forebrain dataset with various dropout rates. The centre line of box plot in a violin plot denotes the median sequencing depth. The lower and upper hinges (bounds of box) correspond to the first and third quartiles (the 25-th and 75-th percentiles). The upper whisker extends from the hinge to the highest value that is within $1.5 \times$ IQR of the hinge, where IQR is the inter-quartile range, or distance between the first and third quartiles. The lower whisker extends from the hinge to the lowest value within $1.5 \times$ IQR of the hinge. Data beyond the end of the whiskers, i.e. the maxima and the minima, are outliers and plotted as points.

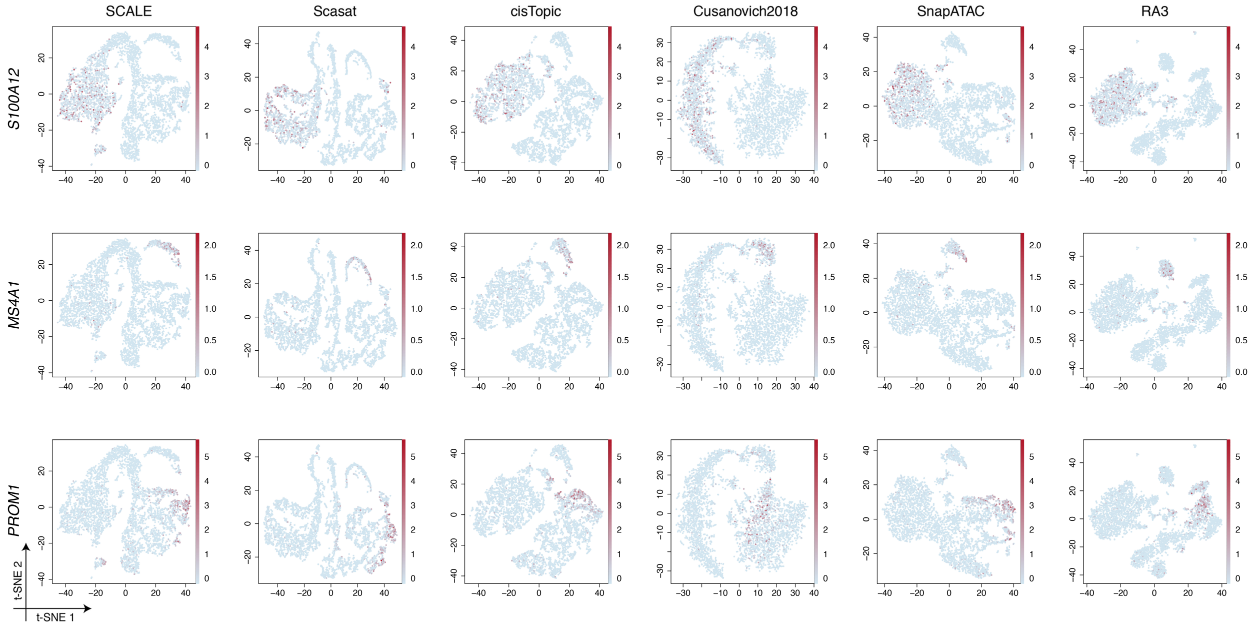

Supplementary Fig. 9 t-SNE visualizations of the cells in the 10X PBMC dataset, using latent features obtained from different methods. Chromatin accessibility of *S100A12* (a marker gene of monocytes), *MS4A1* (a marker gene of B cells), and *PROM1* (a marker gene of CD34+ cells) are projected onto the visualizations, respectively.

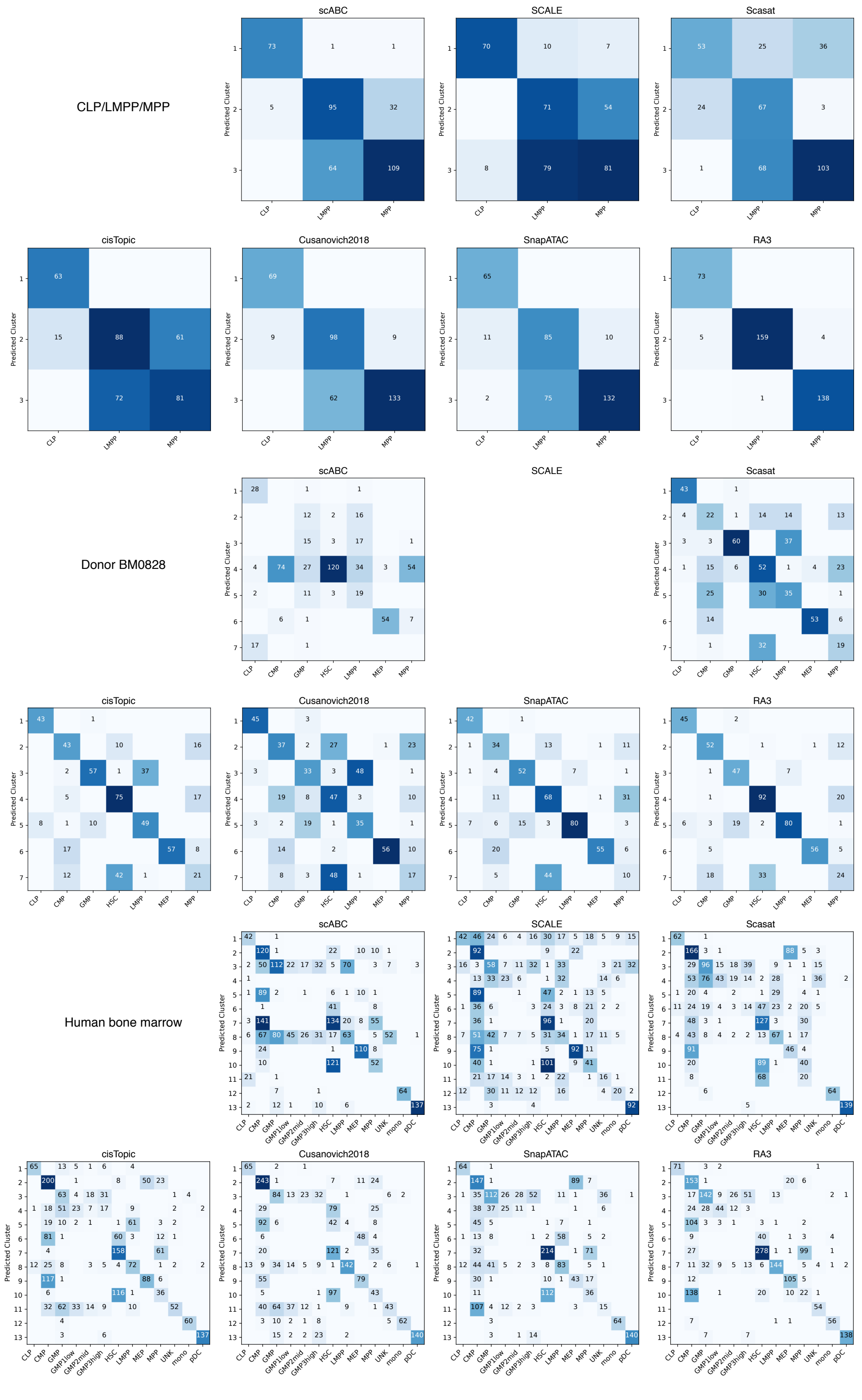

Supplementary Fig. 10 The clustering tables on various datasets using different methods.

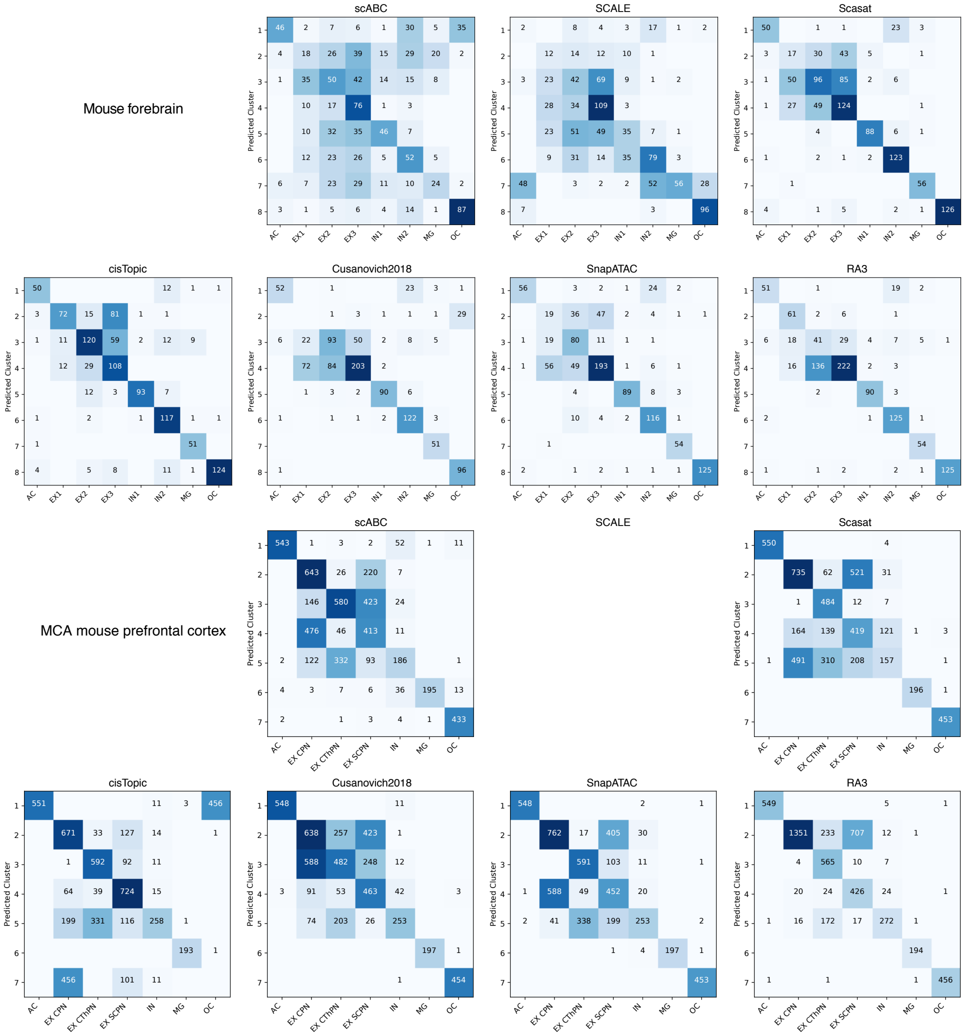

Supplementary Fig. 10 (continue) The clustering tables on various datasets using different methods.

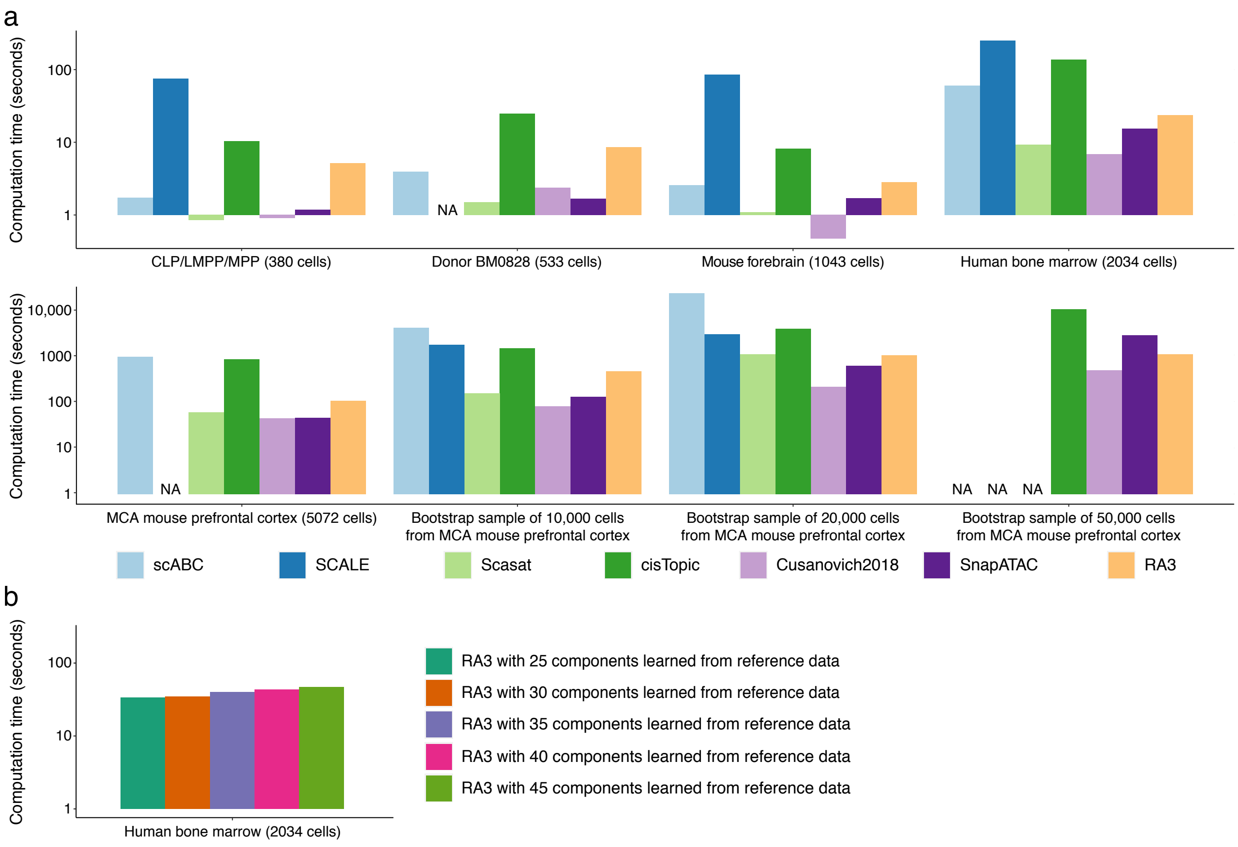

Supplementary Fig. 11 The comparison of computational efficiency and scalability. **a** The computation time of different methods on datasets of various sizes (five real datasets and three datasets simulated by bootstrap on the dataset of MCA mouse prefrontal cortex). For the large dataset with 50,000 cells, scABC did not finish within 1,000 minutes, Scasat exceeds the memory, and SCALE outputs a "Nan" error message. **b** The computation time of RA3 on the human bone marrow dataset using different number of components learned from reference data. The reference data was constructed from BAM files of all the bulk samples in OPENANNO. All the tests were run on a machine with an Intel Xeon E5-2660 v4 X CPU with 14 cores, 4 GeForce GTX 1080 Ti GPUs and 500GB of RAM on the CentOS 7 operating system.

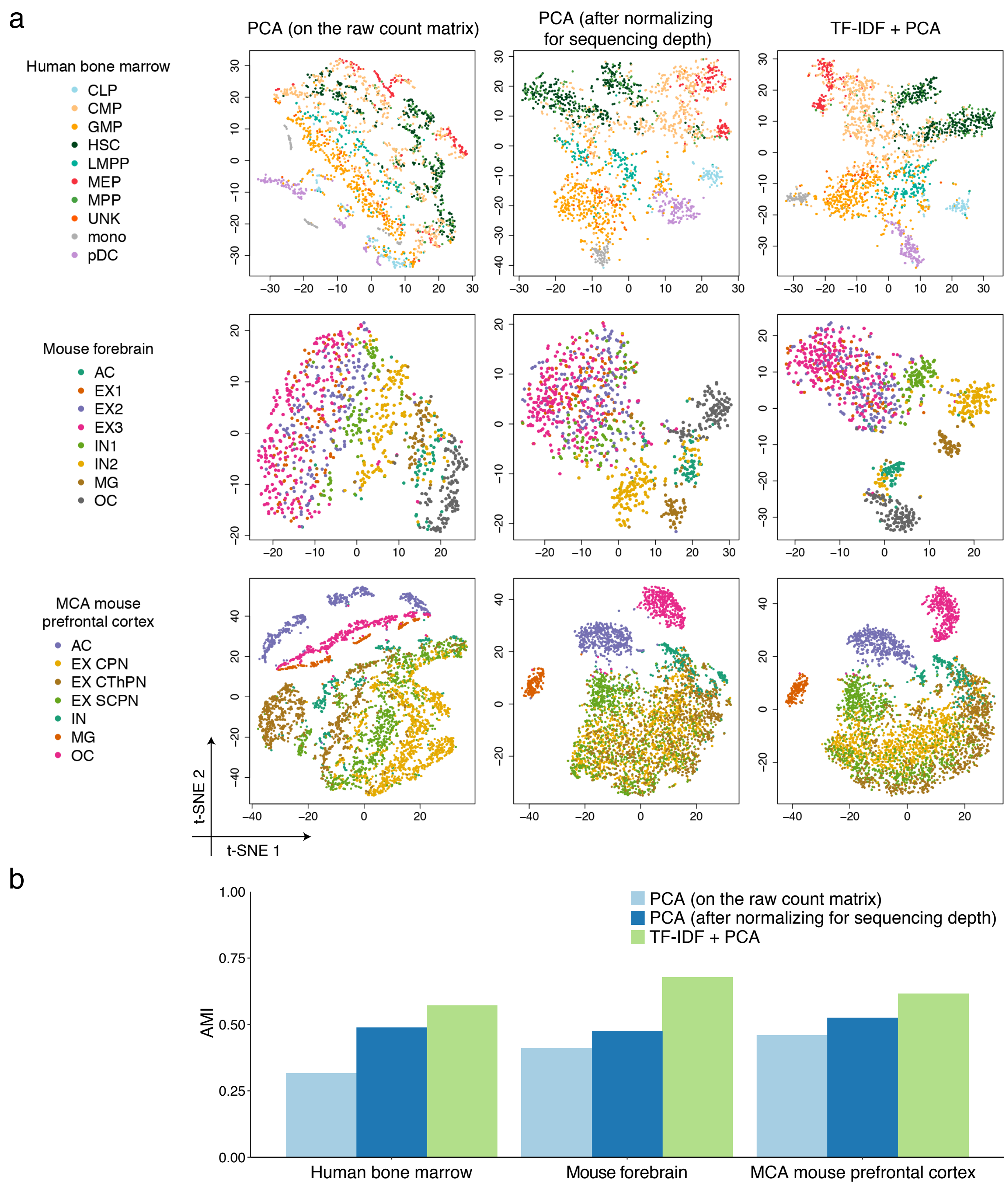

Supplementary Fig. 12 The comparison between PCA (on the raw count matrix), PCA (after normalizing for sequencing depth only) and TF-IDF + PCA. **a** t-SNE visualizations of the cells in different datasets using different methods. **b** The clustering performance using latent features extracted by different methods, evaluated by Adjusted Mutual Information (AMI). Abbreviations: TF-IDF, term frequency-inverse document frequency transformation; PCA, principal component analysis.

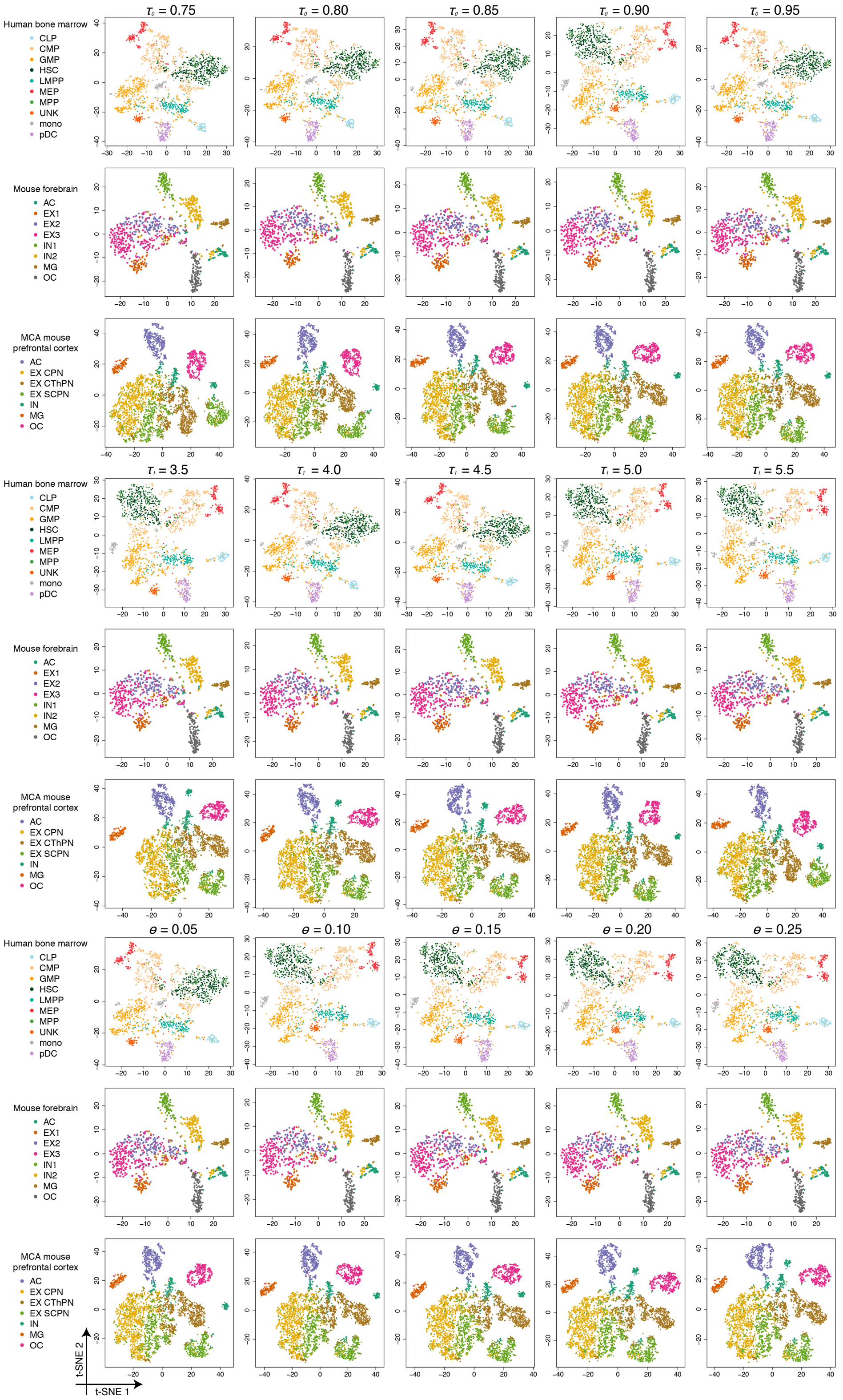

Supplementary Fig. 13 The robustness of RA3 with complete reference to the choice of τ_0, τ_1, θ, K_2 and K_3. When we varied one parameter, we fixed the other parameters to the default setting.

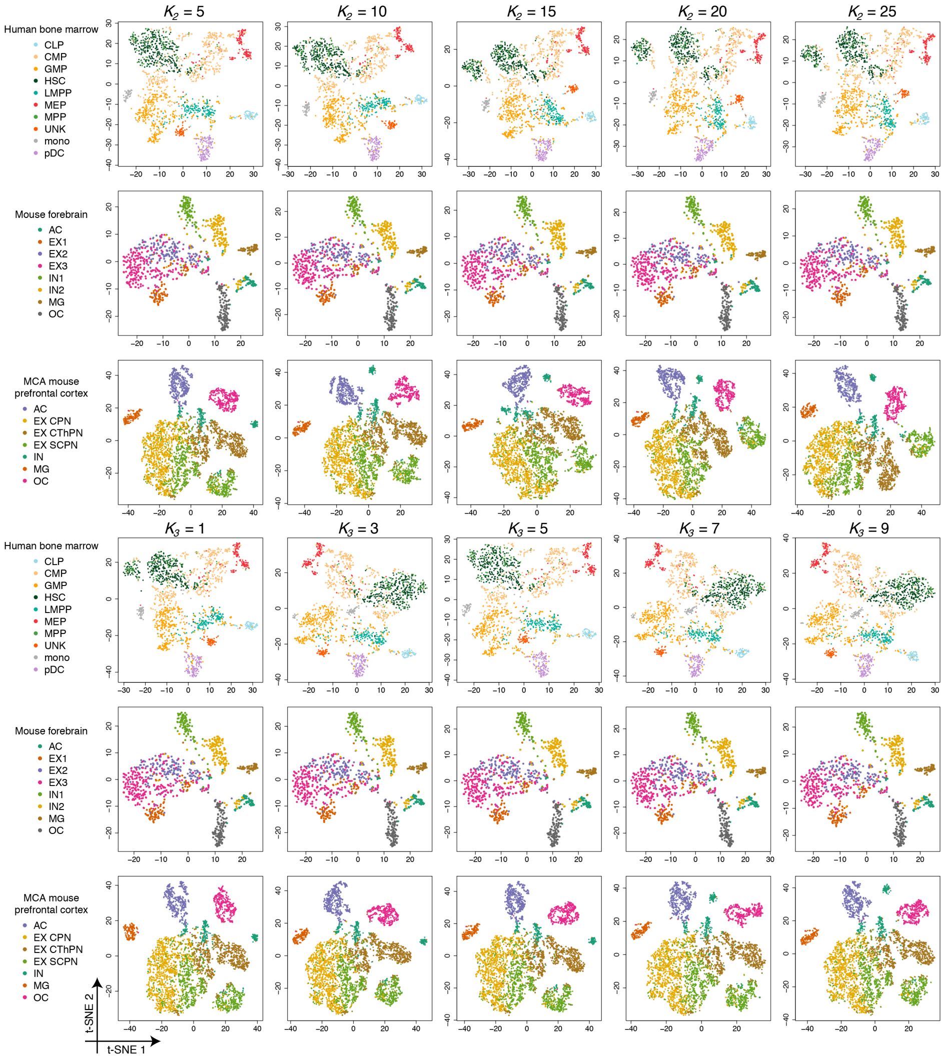

Supplementary Fig. 13 (continue) The robustness of RA3 with complete reference to the choice of τ_0, τ_1, θ, K_2 and K_3. When we varied one parameter, we fixed the other parameters to the default setting.

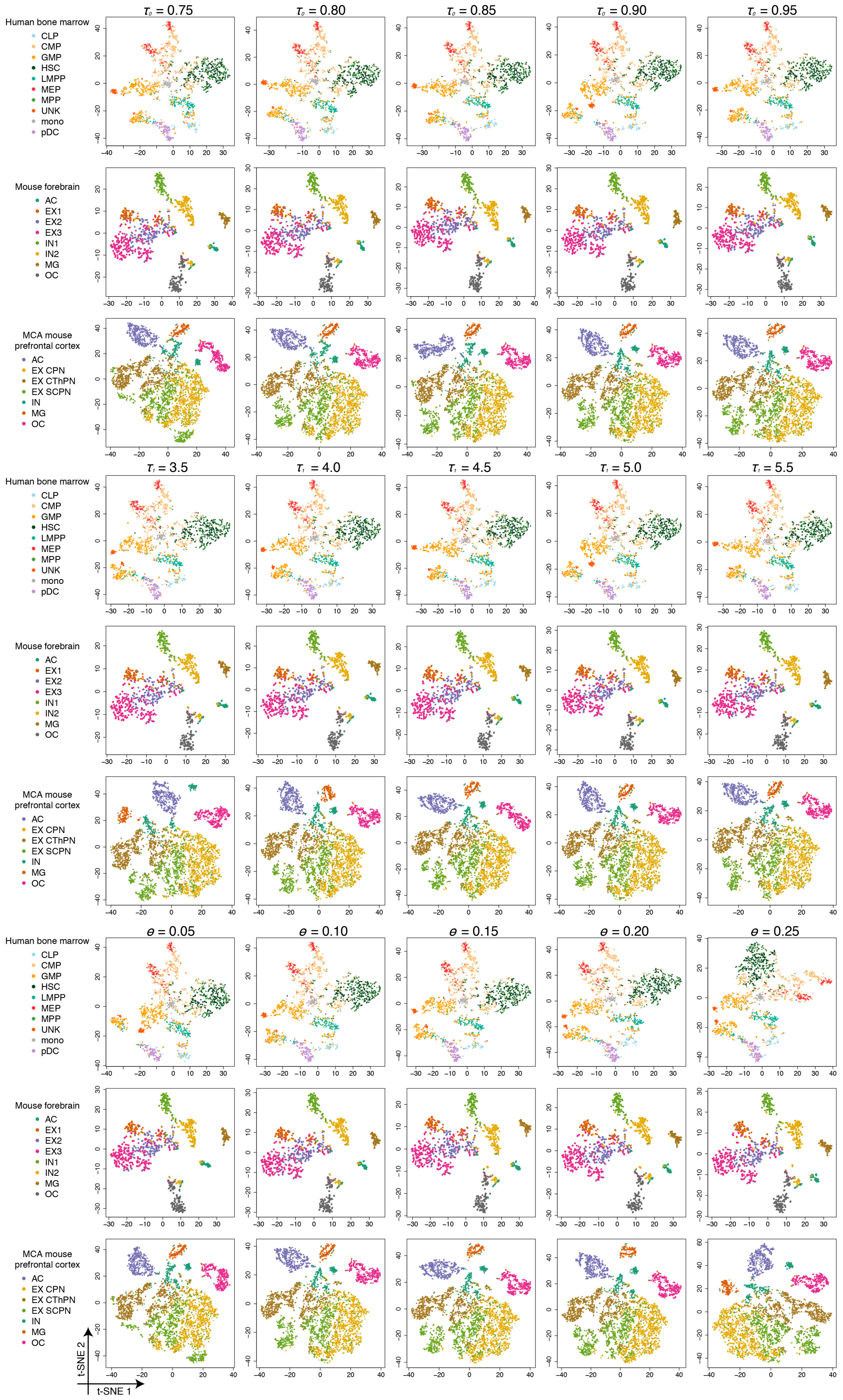

Supplementary Fig. 14 The robustness of RA3 with incomplete reference to the choice of τ_0, τ_1, θ, K_2 and K_3. When we varied one parameter, we fixed the other parameters to the default setting.

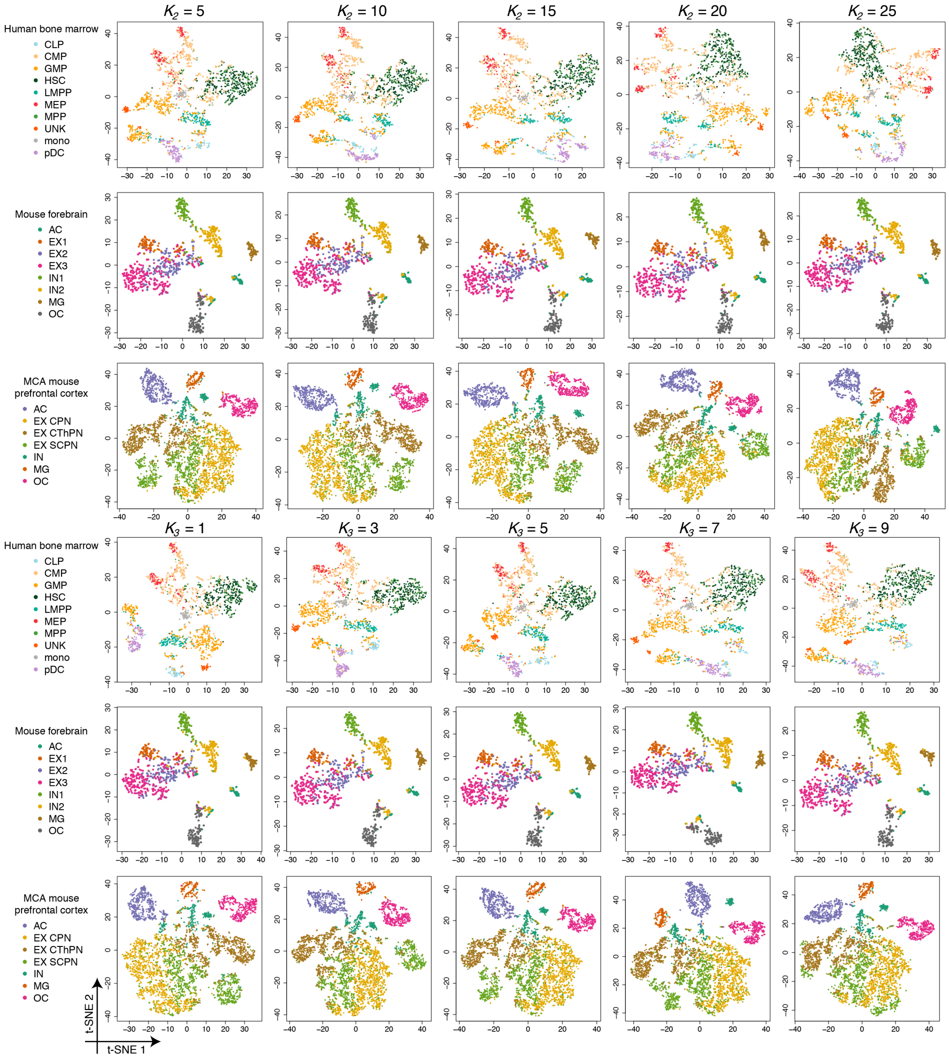

Supplementary Fig. 14 (continue) The robustness of RA3 with incomplete reference to the choice of τ_0, τ_1, θ, K_2 and K_3. When we varied one parameter, we fixed the other parameters to the default setting.

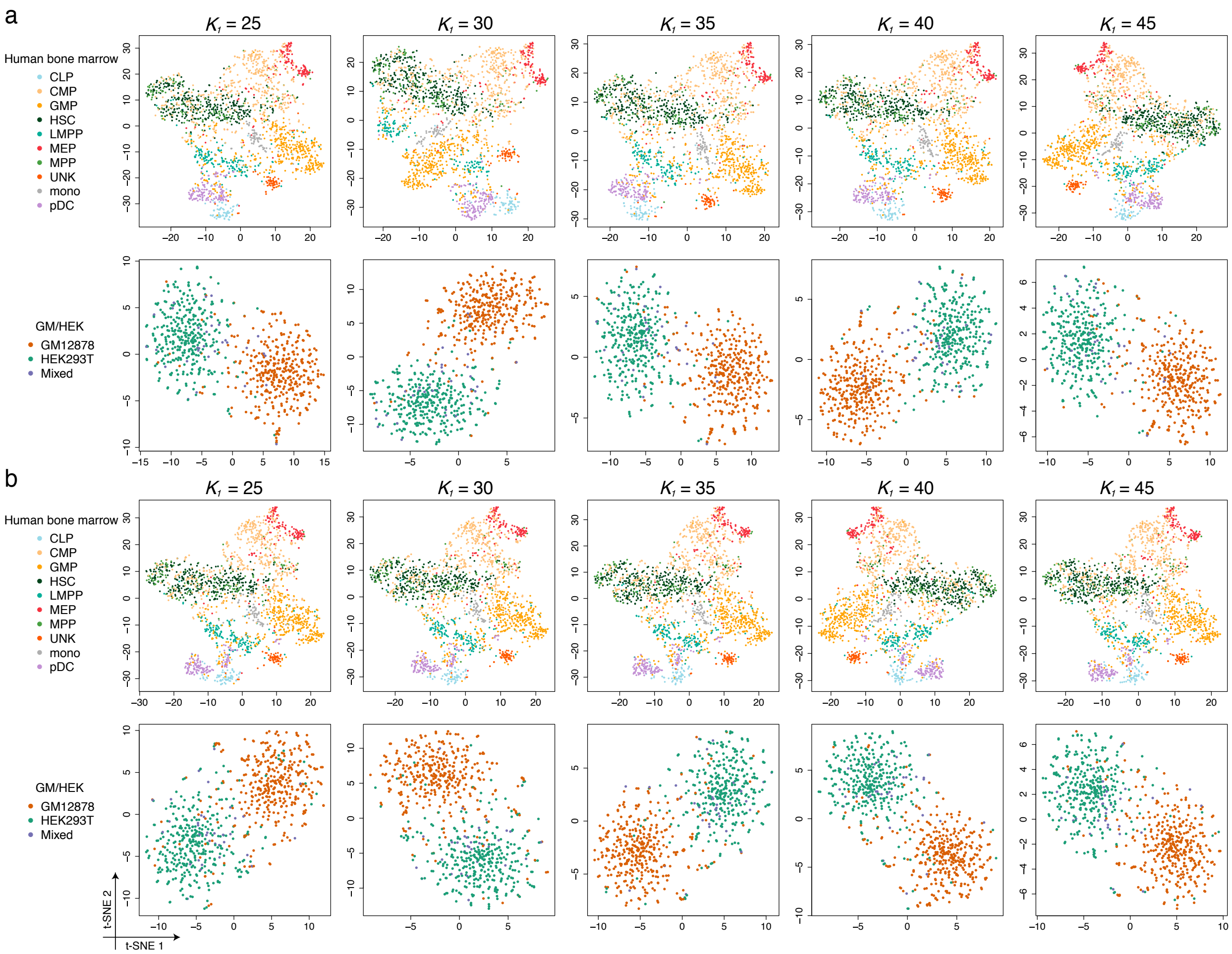

Supplementary Fig. 15 The robustness of RA3 to the choice of number of PCs learned from the reference data. When we varied the parameter, we fixed other parameters to the default setting. **a** t-SNE visualization of the cells in different datasets using latent features obtained from RA3 with reference constructed from BAM files of all the bulk samples in OPENANNO. **b** t-SNE visualization of the cells in different datasets using latent features obtained from RA3 with reference constructed from BED files of all the bulk samples in OPENANNO.

Supplementary Table 1 Identified significant pathways in the GREAT analysis for the second component in RA3, using one-sided binomial test and false discovery rate (FDR) correction.

Term name	Binom raw P-Val	Binom FDR Q-Val
regulation of lymphocyte activation	1.83E-09	4.8042E-06
Fc receptor signaling pathway	7.17E-09	1.3472E-05
immune response-regulating cell surface receptor signaling pathway	1.12E-08	1.6404E-05
immune response-activating cell surface receptor signaling pathway	2.13E-08	2.5471E-05
positive regulation of lymphocyte activation	2.03E-07	0.00014791
negative regulation of mitotic cell cycle phase transition	4.59E-07	0.00023211
negative regulation of cell cycle phase transition	7.71E-07	0.00032686
positive regulation of leukocyte cell-cell adhesion	9.46E-07	0.0003554
stimulatory C-type lectin receptor signaling pathway	1.281E-06	0.00044312
positive regulation of T cell activation	1.3431E-06	0.0004527
innate immune response activating cell surface receptor signaling pathway	1.6952E-06	0.00055707
regulation of transcription from RNA polymerase II promoter in response to hypoxia	5.4868E-06	0.00144247
B cell activation	5.8097E-06	0.00144092
regulation of protein modification by small protein conjugation or removal	6.1286E-06	0.00149186
positive regulation of nuclear-transcribed mRNA catabolic process, deadenylation-dependent decay	6.9655E-06	0.00160635
positive regulation of protein modification by small protein conjugation or removal	1.1139E-05	0.00228792
Fc-gamma receptor signaling pathway involved in phagocytosis	1.1282E-05	0.00224698
regulation of lymphocyte differentiation	1.1859E-05	0.00229239
negative regulation of G2/M transition of mitotic cell cycle	1.2156E-05	0.00228273
Fc-gamma receptor signaling pathway	1.3032E-05	0.00241284
positive regulation of protein ubiquitination	1.5601E-05	0.00273427
negative regulation of cell cycle G2/M phase transition	1.779E-05	0.00299805
Fc receptor mediated stimulatory signaling pathway	1.8549E-05	0.0029377
regulation of transcription from RNA polymerase II promoter in response to stress	2.0917E-05	0.00319709
antigen receptor-mediated signaling pathway	2.2557E-05	0.00336951
regulation of protein ubiquitination	2.3071E-05	0.00336966
vascular endothelial growth factor receptor signaling pathway	2.483E-05	0.00358669
positive regulation of actin filament polymerization	2.8003E-05	0.00391592
negative regulation of homeostatic process	3.0866E-05	0.00414013
T cell receptor signaling pathway	3.6664E-05	0.00472502
SCF-dependent proteasomal ubiquitin-dependent protein catabolic process	4.0321E-05	0.00509631
phagocytosis	4.7166E-05	0.00584906
production of molecular mediator of immune response	6.2411E-05	0.0075265
positive regulation of protein ubiquitination involved in ubiquitin-dependent protein catabolic process	6.5115E-05	0.00771113
regulation of B cell proliferation	7.4386E-05	0.00828648
regulation of stem cell differentiation	8.0264E-05	0.0087196
immunoglobulin production	8.0644E-05	0.00868902
Fc-epsilon receptor signaling pathway	0.00010227	0.01010824
regulation of B cell activation	0.00010539	0.01026214
regulation of DNA-templated transcription in response to stress	0.00011276	0.01066321
innate immune response-activating signal transduction	0.00011788	0.01091213
regulation of protein ubiquitination involved in ubiquitin-dependent protein catabolic process	0.00011816	0.01086189
peptidyl-tyrosine autophosphorylation	0.00025403	0.0183472
signal complex assembly	0.00056481	0.03214032
positive regulation of ubiquitin-protein transferase activity	0.00056821	0.03219435
positive regulation of endothelial cell chemotaxis by VEGF-activated vascular endothelial growth factor receptor signaling pathway	0.00091295	0.04494659
positive regulation of B cell proliferation	0.00109505	0.04998062

Supplementary Table 2 The identified hematopoietic development-associated TF binding motifs.

TF binding motifs	Reference
CTCF	Herold et al., 2012[8]
EBF1	Zandi et al., 2008[9], Somasundaram et al., 2015[10]
LHX6	Poulos et al., 2015[11]
SPI1	Nerlov and Graf, 1998[12]
SNAI2	Pioli et al., 2013[13]
ID4	Martin et al., 2008[?]
TCF3	Somasundaram et al., 2015[10]
TCF4	Somasundaram et al., 2015[10], Buenrostro et al., 2018[1]
FIGLA	Virant-Klun, 2016[14]
ZEB1	Schep et al., 2017[15]
CEBPA	Leroy et al., 2005[16], Lou, 2013[17]
SPIC	Ciau-Uitz et al., 2013[18]
ETV6	Hock et al., 2004[19]
ELF5	Yamamizu et al., 2013[20], Mabbott et al., 2010[21]
SPIB	Mabbott et al., 2010[21]
ETV2	van Bueren and Black, 2012[22]
IRF8	Satpathy et al., 2012[23]
IRF7	Ning et al., 2011[24]
PKNOX2	Cagnan et al., 2019[?]
PKNOX1	Di Rosa et al., 2007[25]
TGIF2	Sugimura et al., 2017[26]
ATF4	Zhao et al., 2015[27]
REL	Mabbott et al., 2010[21]
RELA	Stein and Baldwin, 2013[28]
NFKB1	De Molfetta et al., 2010[29]
NFKB2	De Molfetta et al., 2010[29]
CEBPG	Lou, 2013[17]
CEBPE	Lou, 2013[17], Buenrostro et al., 2018[1]
CEBPB	Lou, 2013[17], Satoh et al., 2017[30]
CEBPD	Lou, 2013[17], Buenrostro et al., 2018[1]
JDP2	Ji et al., 2010[31]
NFE2	Gasiorek and Blank, 2015[32]
FOS::JUN	Liebermann et al., 1998[33]
FOS	Shafarenko et al., 2004[34]
JUND	Liebermann and Hoffman, 2002[35]
JUNB	Liebermann and Hoffman, 2002[35]
GATA1::TAL1	Pimkin et al., 2014[36]
GATA2	Iwasaki et al., 2003[37]
GATA3	Iwasaki et al., 2003[37]

References

1. Buenrostro, J. D. et al. Integrated single-cell analysis maps the continuous regulatory landscape of human hematopoietic differentiation. *Cell* **173**, 1535–1548 e16 (2018).

2. Li, H. et al. The sequence alignment/map format and samtools. *Bioinformatics* **25**, 2078–9 (2009).

3. Li, H. Aligning sequence reads, clone sequences and assembly contigs with bwa-mem. *arXiv preprint arXiv:1303.3997* (2013).

4. Harris, C. R. et al. Array programming with numpy. *Nature* **585**, 357–362 (2020).

5. Virtanen, P. et al. Scipy 1.0: fundamental algorithms for scientific computing in python. *Nat Methods* **17**, 261–272 (2020).

6. McKinney, W. Data structures for statistical computing in python. In *Proceedings of the 9th Python in Science Conference*, vol. 445, 51–56 (Austin, TX).

7. Pedregosa, F. et al. Scikit-learn: Machine learning in python. *Journal of machine learning research* **12**, 2825–2830 (2011).

8. Herold, M., Bartkuhn, M. & Renkawitz, R. Ctcf: insights into insulator function during development. *Development* **139**, 1045–1057 (2012).

9. Zandi, S. et al. Ebf1 is essential for b-lineage priming and establishment of a transcription factor network in common lymphoid progenitors. *The Journal of Immunology* **181**, 3364–3372 (2008).

10. Somasundaram, R., Prasad, M. A., Ungerbäck, J. & Sigvardsson, M. Transcription factor networks in b-cell differentiation link development to acute lymphoid leukemia. *Blood, The Journal of the American Society of Hematology* **126**, 144–152 (2015).

11. Poulos, M. G. et al. Vascular platform to define hematopoietic stem cell factors and enhance regenerative hematopoiesis. *Stem cell reports* **5**, 881–894 (2015).

12. Nerlov, C. & Graf, T. Pu. 1 induces myeloid lineage commitment in multipotent hematopoietic progenitors. *Genes & development* **12**, 2403–2412 (1998).

13. Pioli, P. D., Dahlem, T. J., Weis, J. J. & Weis, J. H. Deletion of snai2 and snai3 results in impaired physical development compounded by lymphocyte deficiency. *PloS one* **8** (2013).

14. Virant-Klun, I. Very small embryonic-like stem cells: a potential developmental link between germinal lineage and hematopoiesis in humans. *Stem cells and development* **25**, 101–113 (2016).

15. Schep, A. N., Wu, B., Buenrostro, J. D. & Greenleaf, W. J. chromvar: inferring transcription-factor-associated accessibility from single-cell epigenomic data. *Nature methods* **14**, 975–978 (2017).

16. Leroy, H. et al. Cebpa point mutations in hematological malignancies. *Leukemia* **19**, 329–334 (2005).

17. Lou, Y.-j. Cebpa-cebpg axis as a novel promising therapeutic target in acute myeloid leukemia. *Acta Pharmacologica Sinica* **34**, 185–186 (2013).

18. Ciau-Uitz, A., Wang, L., Patient, R. & Liu, F. Ets transcription factors in hematopoietic stem cell development. *Blood Cells, Molecules, and Diseases* **51**, 248–255 (2013).

19. Hock, H. et al. Tel/etv6 is an essential and selective regulator of adult hematopoietic stem cell survival. *Genes & development* **18**, 2336–2341 (2004).

20. Yamamizu, K. et al. Identification of transcription factors for lineage-specific esc differentiation. *Stem cell reports* **1**, 545–559 (2013).

21. Mabbott, N. A., Baillie, J. K., Hume, D. A. & Freeman, T. C. Meta-analysis of lineage-specific gene expression signatures in mouse leukocyte populations. *Immunobiology* **215**, 724–736 (2010).

22. van Bueren, K. L. & Black, B. L. Regulation of endothelial and hematopoietic development by the ets transcription factor etv2. *Current opinion in hematology* **19**, 199–205 (2012).

23. Satpathy, A. T., Wu, X., Albring, J. C. & Murphy, K. M. Re (de) fining the dendritic cell lineage. *Nature immunology* **13**, 1145 (2012).

24. Ning, S., Pagano, J. & Barber, G. N. Irf7: activation, regulation, modification and function. *Genes & Immunity* **12**, 399–414 (2011).

25. Di Rosa, P. et al. The homeodomain transcription factor prep1 (pknox1) is required for hematopoietic stem and progenitor cell activity. *Developmental biology* **311**, 324–334 (2007).

26. Sugimura, R. et al. Haematopoietic stem and progenitor cells from human pluripotent stem cells. *Nature* **545**, 432–438 (2017).

27. Zhao, Y. et al. Atf4 plays a pivotal role in the development of functional hematopoietic stem cells in mouse fetal liver. *Blood, The Journal of the American Society of Hematology* **126**, 2383–2391 (2015).

28. Stein, S. J. & Baldwin, A. S. Deletion of the nf-kb subunit p65/rela in the hematopoietic compartment leads to defects in hematopoietic stem cell function. *Blood, The Journal of the American Society of Hematology* **121**, 5015–5024 (2013).

29. De Molfetta, G. A. et al. Role of nfkb2 on the early myeloid differentiation of cd34+ hematopoietic stem/progenitor cells. *Differentiation* **80**, 195–203 (2010).

30. Satoh, T. et al. Identification of an atypical monocyte and committed progenitor involved in fibrosis. *Nature* **541**, 96–101 (2017).

31. Ji, H. et al. Comprehensive methylome map of lineage commitment from haematopoietic progenitors. *Nature* **467**, 338–342 (2010).

32. Gasiorek, J. J. & Blank, V. Regulation and function of the nfe2 transcription factor in hematopoietic and non-hematopoietic cells. *Cellular and molecular life sciences* **72**, 2323–2335 (2015).

33. Liebermann, D., Gregory, B. & Hoffman, B. Ap-1 (fos/jun) transcription factors in hematopoietic differentiation and apoptosis. *International journal of oncology* **12**, 685–1385 (1998).

34. Shafarenko, M., Amanullah, A., Gregory, B., Liebermann, D. A. & Hoffman, B. Fos modulates myeloid cell survival and differentiation and partially abrogates the c-myc block in terminal myeloid differentiation. *Blood* **103**, 4259–4267 (2004).

35. Liebermann, D. A. & Hoffman, B. Myeloid differentiation (myd) primary response genes in hematopoiesis. *Oncogene* **21**, 3391–3402 (2002).

36. Pimkin, M. et al. Divergent functions of hematopoietic transcription factors in lineage priming and differentiation during erythro-megakaryopoiesis. *Genome research* **24**, 1932–1944 (2014).

37. Iwasaki, H. et al. Gata-1 converts lymphoid and myelomonocytic progenitors into the megakaryocyte/erythrocyte lineages. *Immunity* **19**, 451–462 (2003).
